# Experimental Models of Oral Biofilms Developed on Inert Substrates: A Review of the Literature

**DOI:** 10.1155/2016/7461047

**Published:** 2016-09-06

**Authors:** Lopez-Nguyen Darrene, Badet Cecile

**Affiliations:** ^1^Université de Bordeaux, 16 Cours de la Marne, 33082 Bordeaux, France; ^2^ISVV, EA 4577 Unité de Recherche Oenologie, Institut Polytechnique de Bordeaux, Université de Bordeaux, 33140 Villenave d'Ornon, France

## Abstract

The oral ecosystem is a very complex environment where more than 700 different bacterial species can be found. Most of them are organized in biofilm on dental and mucosal surfaces. Studying this community is important because a rupture in stability can lead to the preeminence of pathogenic microorganisms, causing dental decay, gingivitis, or periodontitis. The multitude of species complicates biofilm analysis so its reproduction, collection, and counting are very delicate. The development of experimental models of dental biofilms was therefore essential and multiple* in vitro* designs have emerged, each of them especially adapted to observing biofilm formation of specific bacteria within specific environments. The aim of this review is to analyze oral biofilm models.

## 1. Introduction

The oral cavity is a complex environment harboring more than 700 bacterial taxa. One major player in this ecosystem is dental plaque which develops naturally on hard and soft tissues of the mouth. Most oral bacteria are found in this biofilm whose complex organization remains relatively stable over time despite regular environmental changes [1–4]. Pathologies such as dental caries or periodontitis may arise when the equilibrium is compromised and when an imbalance occurs among the indigenous bacteria [5].

For many years, the oral ecosystem was studied with a reductionist approach, microbiologists studying bacterial species individually. This strategy made it possible to review and understand all the different components of this ecosystem, but without being able to explain how bacteria can form biofilms or to understand their functioning. The development of experimental models of dental biofilms was therefore essential and multiple* in vitro* models have emerged, each of them especially adapted to observing biofilm formation of specific bacteria within specific environments.

The aim of this review is to present currently available oral biofilm models. Various experimental designs have been developed from simple ones with a single bacterium to more complex multispecies designs.

Interests and limits of each model described below are given in Table 1.

## 2. Saliva and Medium

### 2.1. Saliva

Adhesion of bacteria to solid substratum is often mediated by a conditioning film of molecules adsorbed to the surface. In the oral cavity, the dental pellicle needs to be deposited on tooth surfaces for oral biofilm to develop. It is mostly composed of salivary proteins.

In order to mimic this coat, some authors recommend using artificial saliva, the major advantage being that it is reproducible.

Pratten compared various artificial saliva compositions: basic saliva first described by Russell and Coulter [6], hybrid saliva (with modified proportions), modified saliva (without lab-lemco), and complete saliva (with more mucins). Complete saliva seems to be the most reasonable compromise [7]. Basic saliva has also been used in other works that aimed to test the effect of antimicrobial agents on orthodontic bonding materials [8], the effect of manganese on* Streptococcus mutans* biofilm [9], or the effect of various oral rinses on the detachment of an artificial oral biofilm [10]. Wong and Sissons compared two different types of saliva: BMM (Basal Medium Mucin) and DMM (Defined Medium Mucin) [11]. BMM is a complex mucin-containing but chemically undefined medium, while DMM is based on the Shellis artificial saliva [12] and contains various ions, vitamins, amino acids, and growth factors at the same rate as in human saliva. Other authors also supplied their biofilm with DMM in order to test the effect of nutrient variations on the formation of biofilms [12–15].

All these artificial media have a simpler composition than natural human saliva. Particularly, they do not contain the various proteins present in the acquired pellicle (e.g., histatins, proline rich proteins) which play an important role in the mechanisms of bacterial adherence. For this reason, human saliva was used in many other studies in order to be closer to oral conditions [16–18]. Human saliva can be collected from only one or several healthy volunteers [19, 20]. It is obtained by splitting at least 1 hour and a half after eating, drinking, and tooth cleaning. Samples are pooled and centrifuged, and the supernatants are pasteurized and recentrifuged before being stored at −20°C [21].

In order to grow biofilms, media have to reach all the complex nutritional requirements to allow the growth of bacteria. Saliva only or its combination with selective media can be used. Regarding selective media, in case of mono-species biofilms, each bacterium has its preferred medium that eases its growth.

In case of plurispecies biofilms, the Fluid Universal Medium, described by Guggenheim et al. [21], can generally allow the growth of many bacterial species, so it has been used as a support for multispecies biofilms. This FUM went through modifications and created the modified FUM (supplemented with 67 mmol/L Sorensen's buffer, pH 7.2), the enriched FUM (+0.15% sucrose, 0.15% glucose). 50% heat inactivated horse serum can be added to help the growth of certain bacteria, as well as N-acetylmuramic acid for* T. forsythia*, of 0.34 mM hemin for* P. gingivalis* [22].

## 3. Substrates

### 3.1. Experimental Oral Biofilms Are Developed on Various Supports

#### 3.1.1. Glass Surfaces

 Hamada and Torii described a very simple device for testing biofilm formation on an inert surface [23]. Briefly, an overnight culture was added to a glass tube containing specific medium and sucrose 1%. The cultures were incubated at 37°C with an angle of 30 degrees. Biofilm formation was evaluated after 24 to 48 hours with the Murchison scale from 0 (no adhesion) to 4 (strongly adhesive) [24]. Hasan et al. used this support to study the effect on sucrose-dependent and sucrose-independent adherence of* S*.* mutans* and the inhibitory effect of a plant extract on these bacteria [25, 26].

This model also enabled the investigation of the adherence capacities of oral lactobacilli for potential probiotic purposes [27] and the antiadherence properties of polyphenolic compounds on oral bacteria [28]. However, this design does not include the formation of the acquired pellicle: the bacteria directly adhere on the glass surface. For the authors, the ability of* S. mutans *cells to colonize various smooth surfaces may be due to the insoluble glucans synthesized from sucrose by the bound glucosyltransferase. Therefore, this experimental model makes it possible to quickly screen the biofilm formation capacity of various strains that possess this enzyme.

#### 3.1.2. Dentin

Most studies carried out on dentin have focused on endodontic infection. Endodontic disease is a biofilm-mediated infection in which* Enterococcus faecalis* is commonly found [29]. The dentin discs used can be of human [30–32] or bovine origin [33–36]. Some other studies have also been performed on human whole teeth [37]. Many studies aimed to evaluate the antimicrobial activity of various solutions and their capacity to eradicate* E. faecalis* biofilm [30, 31, 34, 36]. Unlike the above-mentioned studies, Li et al. worked on the dentin-composite interface subjected to multispecies biofilm [35]. Bovine dentin discs have also been used in a continuous culture model to study the effects of shiitake mushrooms on biofilms composition and cariogenic properties [33].

#### 3.1.3. Enamel

Enamel is mostly used as a substratum for cariogenic biofilm models. Like dentin, it may be of human or bovine origin [38, 39]. The role of sucrose as a cariogenic molecule has been widely investigated using this substratum in batch models [39, 40] or in an artificial mouth [38].

#### 3.1.4. Polystyrene Surfaces

Polystyrene microtiter plates provide a convenient and sterile abiotic surface for studying bacterial biofilm formation. Loo et al. used this support to study* Streptococcus gordonii* biofilm and particularly to identify the genes that code for biofilm phenotypes [41]. Oettinger-Barak et al. as well as Izano et al. used static 96-well plates to investigate the effect of antibiotics on biofilm formation [42, 43]. The biofilms were highlighted with crystal violet staining after a 24-hour incubation. To analyze the effect of the xylitol and ursolic combination or a synthetic peptide, 24-well plates were used to grow biofilms of various* Streptococcus* species [44, 45]. Other species have also been investigated using this medium: for example,* Actinomyces naeslundii* [46] and* E. faecalis* [47]. A comparison between mono-species and duo-species biofilm combining* S. mutans* and* Veillonella parvula* was made by Kara et al. on 96-well plates [48].

In all these studies, bacteria adhered directly on polystyrene surfaces. Other authors have used microtiter plates coated with various substrates. Human saliva was found to allow the growth of mono-species biofilms [49]. Saito et al. inoculated periapical microorganisms on plates coated with collagen to confirm the stimulation of* Fusobacterium nucleatum* biofilm formation by* Porphyromonas gingivalis* [50]. The effect of* Kaempferia pandurata* on multispecies biofilm was investigated by Yanti et al. by coating it on the plates before growing the biofilm [51].

#### 3.1.5. Hydroxyapatite

The use of hydroxyapatite allows studies on synthetic media mimicking dental tissues, thereby avoiding the search for extracted teeth. Many authors have used this medium in form of either beads or discs. Saliva-coated hydroxyapatite beads have been used in various studies. The growth rate and biofilm thickness of a dual biofilm of* S. mutans *and* Streptococcus sobrinus* were studied by Rozen et al. [52]. The adherence properties of bacterial strains as oral probiotic candidates have also been processed on saliva-coated hydroxyapatite beads [53] or discs [27]. Furthermore, hydroxyapatite has been used to investigate the effects of various molecules on* S. mutans* biofilm formation on both beads [50, 54–56] and discs [57–59].

Other authors have investigated dual-species biofilms. Li et al. tested the effect of nicotine on dual-species biofilms of* S*.* mutans* and* Streptococcus sanguinis *[60]. Ali Mohammed et al. worked on the DNase I and proteinase K treatment of* F*.* nucleatum* and* P*.* gingivalis* biofilms [61]. Dual-species biofilms allowed the observation of differences in growth and acid formation between* S. mutans* and* V. parvula* strains [48].

Hydroxyapatite discs were also the medium used in the Zürich model described below [21].

## 4. Incubation Conditions

Bacterial oral biofilm model systems can be divided into two groups: closed batch culture and open continuous culture models.

### 4.1. Batch Models

One commonly used model developed by Guggenheim et al. is called the Zürich model [21]. This multispecies model allows the study of interactions in bacterial communities.

The first version of this model contained five different species (*A. naeslundii*,* Veillonella dispar*,* F. nucleatum*,* S. sobrinus*, and* Streptococcus oralis*). Biofilms are developed on hydroxyapatite discs coated with pasteurized human saliva for 64 hours in anaerobic conditions before collection. This model was subsequently improved by adding more bacterial species [62]. Using this more recent model, Ammann et al. demonstrated the importance of nutritional conditions for biofilm development and brought some changes to the culture conditions. The Zürich model has been used extensively to test the effect of various components like plant extracts, polyphenolic compounds, and mouthwashes [28, 59, 63, 64]. Furthermore, it has been used to study the effect of xylitol on a growing biofilm [65]. While various studies have described biofilm formation in static systems, bacteria in the oral cavity are subject to constantly changing environmental conditions (e.g., saliva flow conditions). Static models are not able to simulate these conditions so dynamic models are required.

### 4.2. Continuous Culture Models

#### 4.2.1. Constant Depth Film Fermenter

The Constant Depth Film Fermenter is a dynamic biofilm model that allows the control of environmental factors such as the substratum, the nutrient source, and the gas flow [66]. Even biofilm thickness can be controlled [67]. Mono-species biofilm can be studied in this apparatus [68], but the principal advantage is to work with multispecies biofilm mimicking* in vivo* conditions as closely as possible. For example, Ready et al. assessed the resistance of a multispecies oral biofilm to tetracycline with this model [69].

The concept consists in a glass cylinder that contains a stainless steel plate linked to an electric motor that allows the plate rotation. Pores at the cylinder summit enable gas and medium to enter and exit. On the plate, wells are dug into which discs or substratum can be dropped. Temperature and gas flow are controlled and medium and saliva are injected with a pump. Excessive medium is absorbed. The Constant Depth Film Fermenter is a complex system allowing only one antimicrobial formula to be tested at a time so it has been improved, and two different treatments can now be performed at the same time [33, 70].

#### 4.2.2. Flow Cell Chamber System

This model consists in a glass slide coated with saliva that is placed in a chamber and is crossed by a continuous flow of medium [71, 72]. Schlafer et al. tested the effect of osteopontin on a multispecies biofilm using this model [73]. Furthermore, by allowing the evaluation of biofilm development under flow and shear conditions, it has been used to assess antibiotics [42]. Periodontal biofilm can also be developed with it [74].

## 5. Biofilm Collection and Analysis

The methods used to identify different microorganisms in a microcosm biofilm vary according to the models. There are two approaches: cultivation-based and non-cultivation-based.

### 5.1. Cultivation-Based Methods

This technique needs the biofilm to be collected. Some authors recommend vigorous vortexing to remove cells from the biofilm [64, 75]. Ready et al. add a sonication step after vortexing the biofilm [69]. Wirtanen et al. harvest the biofilm by scratching the surface of the tray with a swab and then immersing it in a dilution medium [76]. In their Zürich model, Guggenheim et al. scratch the surface of the disc with a sterile curette to harvest all the cells of the biofilm, even those that are firmly attached [21]. The collected biofilm is then plated on various selective agar media. The distinct colony morphology and gram staining allow the species to be differentiated. This technique of counting colony forming units makes it possible to investigate the effect of various components on the viability of bacteria both on mono-species biofilms [44, 45, 56] and on plurispecies ones [63, 65, 77, 78]. However, it is a time-consuming method and noncultivable species cannot be included in the biofilm. Moreover, scratching of biofilms on hydroxyapatite surfaces may not be easily reproducible.

### 5.2. Non-Cultivation-Based Methods

Since oral diseases have a complex etiology and because only around 50% of oral biofilm can be grown at present, culture-independent molecular-based approaches have been developed that give a more comprehensive assessment of the presence of a range of putative pathogens in samples [78]. In studies on* E. faecalis* biofilms, dentin specimens were stained with BacLight and observed with a fluorescence microscope [30]. In multispecies models, fluorescence* in situ* hybridization (FISH) in combination with epifluorescence and confocal laser scanning microscopy (CLSM) are other standard methods for the visualization and identification of species.

#### 5.2.1. *In Situ* Hybridization Fluorescence (FISH)

A sequential FISH approach allows multiple populations to be detected in a biofilm sample [79]. Indeed, FISH is a recognized tool for the specific identification of targeted bacteria within multispecies biofilms [62]. Moreover, Thurnheer et al. showed that it is possible to perform several consecutive FISH procedures with multiple rRNA to identify simultaneously many members of biofilms [80]. FISH can also be combined with CLSM [62, 64].

#### 5.2.2. Epifluorescence Microscopy and Confocal Laser Scanning Microscopy (CLSM)

The LIVE/DEAD® BacLight*™* fluorescence solution can be used to differentiate viable cells from nonviable ones in terms of membrane integrity. Viable cells are stained with SYTO9® which fluoresces green, while the nonviable ones are stained with propidium iodide which fluoresces red. Using BacLight LIVE/DEAD, Standar et al. inspected cells by fluorescence microscopy when they worked on the biofilm behavior of mixed-species cultures with dental and periodontal pathogens [81]. Chávez de Paz also used this technique to assess cell viability within multispecies biofilms in root canals [82].

CLSM has also been widely used to observe biofilms in three dimensions. It allows the systematic collection of high-quality biofilm images suitable for digital image analysis [79]. After 15 mn dark incubation, de Carvalho et al. use an excitation wavelength of 488 nm to collect all light emitted between 500 and 550 nm and over 560 nm by various filters. They use the scan mode time series to take a series of time-lapse scans at intervals of 10 s during 590 s in continuous scanning mode with a 10x objective lens [83]. Hobby et al. incubate the wells for 18 mn before using a Zeiss LSM 510 Meta confocal scanning system [84].

Some models combine non-cultivation-based and cultivation-based methods. According to Blanc et al., it is thus possible to determine the presence of all the species within the biofilm structure, the volume occupied by the bacteria, and the distribution of live and dead cells at the different biofilm development times [85].

#### 5.2.3. Scanning Electron Microscopy (SEM)

Standar et al. use SEM to observe their multispecies biofilms models. Biofilms are fixed for 24 hours in a 2.5% glutaraldehyde solution and the supports are rinsed with 0.1 M Na-acetate buffer and dehydrated with a graded ethanol series. Then they are subjected to critical point drying with CO_2_, covered with gold (10 nm thickness) and examined with a Zeiss DSM 960 A electron microscope [81]. Howlin et al. also use this technique to visualize biofilms after their removal with an ultrasonically activated water stream [86]. Thurnheer et al. also use SEM to study the role of red complex bacteria in the colonization of gingival epithelia by subgingival biofilms* in vitro *[74].

#### 5.2.4. PCR

Until recently, PCR was mostly used to identify and count bacterial species* in vivo* or in dental plaque samples in connection with oral diseases (caries, periodontitis) [87, 88]. However, in more recent studies, it has also been used to identify species in* in vitro* models either after culture or directly within the biofilm. For example, Zaura et al. used quantitative real-time PCR (qPCR) to observe microbial shifts due to the effect of shiitake mushroom on an* in vitro* caries model [33].

In 2013, Ammann at al. compared a qPCR assay with fluorescence microscopy and colony forming unit counting on selective agars. They found that all ten species included in their* in vitro* biofilm were successfully quantified using qPCR and FISH or immunofluorescence as well as the eight species culturable on selective agar plates. They concluded that CFU counts yielded lower values than the other methods. The same authors also used qPCR combined with CLSM following FISH to compare the quantitative distribution of bacteria and the three-dimensional structure of biofilms either with or without early colonizing species added at a later time point [22]. For a very close purpose, Karched et al. using only qPCR showed that six periodontal species were able to form multispecies biofilm up to eight days* in vitro* without pioneer plaque bacteria [89].

The limitation of qPCR is its inability to discriminate between live and dead cells. Extracellular DNA present in the matrix of the biofilm can also be quantified. To overcome this problem, propidium monoazide has been used in association with qPCR [90, 91]. The results of these studies demonstrated the efficiency of PMA for differentiating viable and dead strains of various species.

## 6. Conclusion

Because biofilms constitute a privileged way of life for oral bacteria, a clear understanding of the processes involved in their formation, their pathogenicity, and their resistance in various biocides is essential for their control. While several experimental models have been proposed to date, differences in biofilm formation times, growth media, incubation conditions (static or flow, aerobic or anaerobic), and the procedures for collecting and analyzing biofilms make a comparison difficult. Choosing the most suitable procedure depends on the particular objective that is sought and on the laboratory facilities that are available.

## Figures and Tables

**Table 1:** Interests and limits of various experimental models of biofilms.

	Interest	Limits
Saliva		
Human	(i) Contains a complex & complete blend of proteins, glycosaminoglycans, and ions that form a pellicle on tooth surface	(i) Quality: need healthy volunteers (ii) Quantity: need many volunteers (iii) Limited reproducibility
Artificial	(i) Reproducibility (ii) Low cost	(i) Less complex blend of molecules (ii) Do not mimic *in vivo* conditions

Substrates		
Glass	(i) Allows a simple and fast screening (ii) Low cost	(i) Direct bacterial adherence: no EAP creation (ii) Scoring could be operator dependent
Dentin/enamel	(i) Study of cariogenic, periodontal, endodontic, and Dentin/Composite interface specific biofilms (ii) Close to *in vivo* condition	Need for human or bovine teeth
Polystyrene (96-well plates)	(i) Can be coated with collagen, saliva, and/or different substances (ii) Allows many simultaneous studies: comparison of different stains, media, and substances in the same array	(i) When not coated: only direct bacterial adherence (ii) Far from *in vivo* conditions
Hydroxyapatite	(i) Best synthetic substrate mimicking human dental tissues (ii) Avoid the search of extracted teeth and their sterilization (iii) Can be coated with collagen, saliva & different substances	(i) Cost (ii) When not coated: only direct bacterial adherence (no EAP creation)

Incubation conditions		
Batch models	(i) Multispecies biofilms (ii) Allows the study of interactions in bacterial communities and the effect of various substances	(i) Far from *in vivo* conditions: does not integrate the changing environmental conditions occurring during biofilm growth
Continuous culture		
Constant depth fermentor	(i) Allows the control of environmental factors: gas flow, real time medium and waste monitoring, biofilm thickness, temperature, and pH (ii) Allows the formation of multispecies biofilms	(i) Cost (ii) Complexity of protocol (iii) No vast simultaneous studies allowed (iv) Can only handle up to 2 experiments at a time
Flow cell chamber	(i) Allows the control of environmental factors (ii) Allows real time microscope observation	

Biofilm collection		
Scrapping	Allows the removal of almost all the biofilm	(i) Operator-dependent
Vortexing & sonification	(i) Reproducibility (ii) Fast and easy	The first (deeper) bacterial layer can remain on the medium

Biofilm analysis		
Cultivation on agar media	(i) Simple (ii) Allows further identification methods (iii) Selection of sustainable strains	(i) Delayed results (ii) Only for culturable species (iii) Time consuming
Gram staining	(i) Low cost (ii) Fast and easy	(i) Limited identification based on colony and bacterial morphology
FISH	(i) Can focus on targeted bacteria in a multispecies biofilm (ii) Possible to combine consecutive FISH with multiple rRNA (iii) Can be combined with CLSM and PCR	(i) Cost (ii) Complexity of protocol (iii) Inability to discriminate live and dead bacteria
CLSM	(i) Allows discriminating between live and dead bacteria (ii) Can determine the distribution of all the different species within the biofilm at different development times (iii) Can be combined with FISH and PCR	(i) Cost (ii) Complexity of protocol (iii) Inability to discriminate stains (only on morphology) (iv) Inability to assess gene expression
SEM	(i) Can determine the distribution of all the different species within the biofilm	(i) Cost (ii) Complexity of protocol (iii) Inability to discriminate live and dead bacteria
PCR	(i) Allows identifying and counting bacterial stains directly (ii) Can be combined with culture on specific media FISH: better results than CFU counts CLSM	(i) Cost (ii) Multispecies biofilms need a cultivation and isolation of every colony prior to PCR (iii) Inability to discriminate live and dead bacteria
